# Stress management preferences and stress experiences among Latinx immigrants in the United States during the COVID-19 pandemic: Mixed-methods results from a community-academic research team

**DOI:** 10.1017/gmh.2024.101

**Published:** 2024-10-23

**Authors:** Shanna D. Stryker, Julia Rabin, Stephanie Castelin, Farrah Jacquez, Karen Chinchilla, Jenniffer Peralta, Lisa M. Vaughn

**Affiliations:** 1 Department of Family and Community Medicine, University of Cincinnati College of Medicine, Cincinnati, OH, USA; 2 Department of Psychology, College of Arts & Sciences, University of Cincinnati, Cincinnati, OH, USA; 3 Latinos Unidos por la Salud, Cincinnati, OH, USA; 4 Department of Pediatrics, Cincinnati Children’s Hospital Medical Center, Cincinnati, OH, USA; 5 College of Criminal Justice, Education, and Human Services, University of Cincinnati, Cincinnati, OH, USA

**Keywords:** Latinx, Latine, Hispanic, Stress reduction, Mental health, Resilience

## Abstract

**Background:**

Latinx individuals in the U.S. have higher levels of stress than other ethnic groups. Latinx immigrants living in non-traditional immigration destinations (NTIDs) have worse access to social and medical support and were particularly vulnerable during the COVID-19 pandemic. This study aims to contextualize stress in Latinx immigrants in an NTID during the COVID-19 pandemic and to understand Latinx immigrants’ preferences for stress management interventions given the sociopolitical and public health context.

**Method:**

Using a community-based participatory research approach with mixed methods research design, community co-researchers gathered data using a quantitative survey and then contextualized survey results using a qualitative community conversation.

**Results:**

Community conversation participants were surprised at the relatively low levels of reported stress and pandemic impact in survey participants, and they proposed the reason was the level of pre-pandemic stressors. Guatemalan immigrants in an NTID reported more stigma but fewer changes between pre- and post-pandemic stress levels. Survey respondents preferred to learn about stress management through YouTube videos or groups led by professionals.

**Conclusions:**

Understanding the diversity of stress experiences among Latinx immigrant groups is critical to developing effective interventions. Coping strategy preferences are variable among different Latinx immigration groups, but asynchronous and/or professional-led stress management was preferred.

## Impact statement

This study partnered with community members to study the impact of the COVID-19 pandemic on stress in Latinx immigrants in an area without significant social or medical support for Latinx immigrants. Our results confirmed that the pandemic and stress were experienced differently between subgroups of Latinx immigrants. Guatemalans were more likely to report experiencing stigma or discrimination and were less likely to report an increase in stress during the pandemic. Community conversation participants suggested that this was related to their high levels of pre-pandemic stress and emphasized the need for more resources serving local Guatemalan immigrants. Guatemalans also reported different uses of stress management techniques and were more likely to prefer learning about stress management through text messaging groups or YouTube videos. Previous work from our group showed that stress and stress management are priority health concerns within the Latinx immigrant community in our city, and that peer-led stress management interventions are effective and acceptable. Despite this, only about half of the surveyed participants wanted to learn more about stress management 9–14 months after the start of the COVID-19 pandemic, and most participants preferred to learn about stress management through watching YouTube videos and preferred professional-led groups over peer-led groups. These results are important for showing that when designing programs to serve Latinx immigrants, a one-size-fits-all approach is unlikely to be effective, particularly in areas with limited social and medical support for these communities. In addition, asynchronous methods of teaching about stress management are likely to be preferred.

## Introduction

As of 2020, Latinx individuals are the second-largest racial/ethnic minority group in the U.S., only outnumbered by non-Hispanic White individuals (Funk and Lopez, 2022). Due to sociopolitical and economic factors (including changes to U.S. immigration law), there has been a consistent and rapid growth in the number of immigrants living in the U.S. since 1970 (Valentín-Cortés et al., 2020). In 2018, about half of U.S. immigrants came from Latin America (Budiman et al., 2020). States with the largest number of immigrants include California, Texas, New Jersey, New York, Florida and Nevada (Funk and Lopez, 2022).

Geographic areas outside of these high-volume states, called non-traditional immigration destinations (NTIDs), have experienced an exponential increase in the Latinx immigrant population only in recent years. Because of this, these locations often lack the infrastructure, Spanish language services, and community support to effectively meet the needs of Latinx newcomers (Jacquez et al., 2016). Latinx immigrants living in NTIDs are more likely to struggle with access to health services compared to peers in traditional immigration destinations (Gresenz et al., 2012; Topmiller et al., 2017; Zhen-Duan et al., 2017; Esterline and Batalova, 2022). Additionally, Latinx individuals living in NTIDs are at greater risk of social and economic disadvantages and exclusion (Crowley et al., 2015; Jacquez et al., 2015, 2016).

### Stress among Latinx individuals

Stress is a physiological or psychological response in which a person feels tense, restless, nervous, or unable to relax because their mind feels troubled. Latinx adults have higher stress levels than those reported by other ethnicities (American Psychological Association, 2016; McKnight-Eily et al., 2021). Documented sources of stress among immigrants include ethnic discrimination, concerns over immigration status and family separation, and socioeconomic inequalities (American Psychological Association, 2016; McKnight-Eily et al., 2021). Stress associated with racism and discrimination has been shown to negatively affect mental and physical health in Latinx individuals (Carvajal et al., 2013; Cobb et al., 2021; Paradies et al., 2015; Valentín-Cortés et al., 2020). The health implications of high-stress levels are particularly concerning given that Latinx immigrants in the U.S. have poorer access to health services that could offer treatment (Ortega et al., 2015; Ramos, 2022).

### COVID-19 pandemic

Compounding the existing levels of stress, Latinx communities in the U.S. were disproportionately affected by the COVID-19 pandemic (Webb Hooper et al., 2020). Compared to non-Hispanic White individuals, Latinx individuals were more likely to be diagnosed with and die due to COVID-19, experience financial or job loss, and experience stress due to housing and food instability, especially in NTIDs (Webb Hooper et al., 2020; Collins Niesz, 2021; Hibel et al., 2021; McKnight-Eily et al., 2021; Holden et al., 2022; Martin et al., 2022; Bovell-Ammon et al., 2023). During the pandemic, many people in the U.S. required assistance with basic needs (e.g., healthcare, food), but anti-immigrant legislation such as the “public charge rule” (which stipulated that use of public services by U.S. immigrants could revoke green card eligibility) made immigrants hesitant to access available services (Miller et al., 2020; Page et al., 2020; Wang et al., 2022). Unsurprisingly, mounting evidence suggests these structural and systemic stressors, which were exacerbated during COVID-19, negatively impacted the mental health of Latinx individuals (Swaziek and Wozniak, 2020; Garcini et al., 2021; Gomez-Aguinaga et al., 2021; Hibel et al., 2021; Serafini et al., 2021).

### Current setting

Cincinnati, Ohio, the location for this project, is an NTID. Since 2010, the Latinx population in Cincinnati has grown 48.5%, with Central American immigrants outnumbering other Latinx groups (George Mason University Institute for Immigration Research, 2018; Esterline and Batalova, 2022). In fact, the yearly growth rate of the Latinx population is nearly double the national rate (4.0 vs. 2.2%, respectively) (Hispanic Chamber Cincinnati, 2015). A higher proportion of Latinx individuals in Cincinnati are immigrants than in other similar metro areas due to this recent influx (Hispanic Chamber Cincinnati, 2015). In Cincinnati, the median household income for Latinx households was 85% that of all households in 2020, and the unemployment rate is higher than the overall unemployment rate (Economics Center Research and Consulting, 2022). Based on available data, in 2020, 91.5% of the total Cincinnati population had at least a high school diploma, while 76.8% of the local Latinx population did. The three industries within which the Latinx population is most likely to be employed are manufacturing (14.0%) and administrative, support, waste management, and remediation services (14.0%), and accommodation and food services (11.1%) (Economics Center Research and Consulting, 2022).

Within this context, we aimed to build on prior work that showed that stress is a health priority for the Cincinnati Latinx community (Jacquez et al., 2019). Given that Latinx immigrants in NTIDs face barriers to accessing health services, we aimed for our work to contextualize stress during the COVID-19 pandemic in Latinx immigrants in an NTID and to understand Latinx immigrants’ preferences for stress management interventions given the sociopolitical and public health context.

## Methods

### Research design

**Figure 1.**
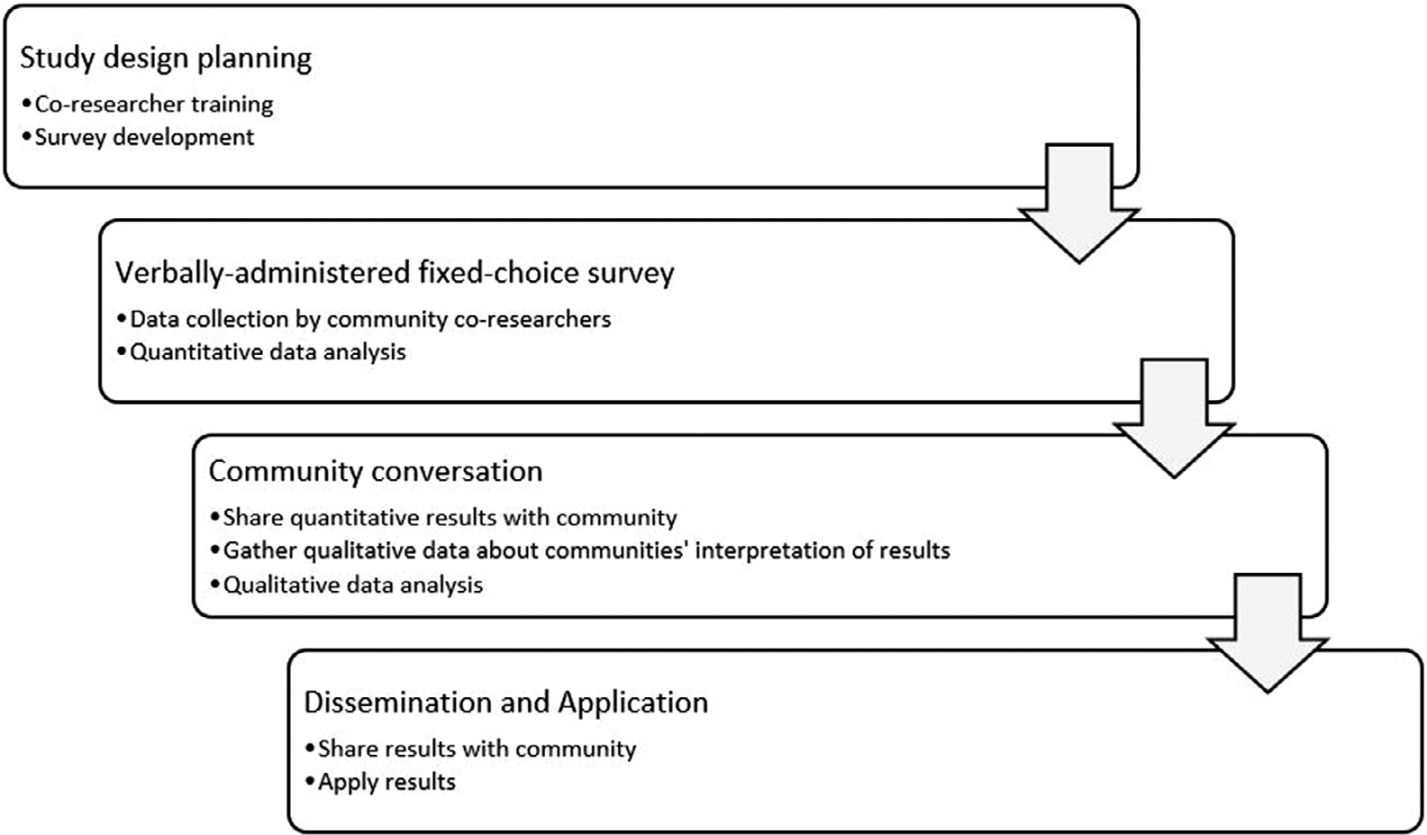
Study design.

Community-based participatory research (CBPR) is a research approach that involves shared decision-making between community members and academic partners and has the goal of generating meaningful knowledge that is responsive to the needs of the community (Israel et al., 1998; Wallerstein and Duran, 2006). Latinos Unidos por la Salud (LU-Salud) is a Cincinnati-based CBPR group focused on addressing Latinx health in an NTID (Vaughn et al., 2016, 2017; Jacquez et al., 2019). LU-Salud is a group of community members that was formed in 2013 with academic partners at the University of Cincinnati (UC) who were involved in community-engaged research to study the health priorities of the local Latinx population. Stress was identified as a community priority in the original survey, and in 2019 an opportunity for additional funding emerged to continue work LU-Salud had been doing with group stress management visits. Upon funding announcement in January 2020, the network of LU-Salud co-researchers was engaged over text asking which members were interested in paid work on this initiative, and two members who had been involved in the original and subsequent studies expressed interest. In line with Israel et al.’s CBPR model for maintaining and sustaining engagement, the two LU-Salud community co-researchers contributed to the study design and were involved with survey development, data collection, qualitative data analysis, and dissemination after receiving training on each of these by the academic team (Israel et al., 1998). This study applied CBPR principles within a mixed methods design, the phases of which are illustrated in Figure 1.

### Procedures

This study was reviewed by the Institutional Review Board of the University of Cincinnati (UC) and determined to be exempt from ongoing oversight given its low risk, lack of collection of identifying information, and goal of informing local initiatives. After survey development, community co-researchers recruited adult Latinx immigrants in Cincinnati between December 2020 and May 2021 via social media advertisement and their social networks and verbally administered the survey during this same period in Spanish (or Mam when necessary). Regular meetings between community co-researchers and academic partners occurred to solve difficulties with recruitment and COVID-19 safety protocols. Survey responses were recorded on printed interview guides and later entered into Research Electronic Data Capture (REDCap) by academic partners for data analysis. Manual double data entry was done on 15% of surveys for data verification (Barchard and Verenikina, 2013). Surveys were completed either via telephone conversation or in person. Participants were given $5 gift cards after survey completion.

### Participants

Survey participants included 121 adults, the average age of whom was 36.62 (*SD* = 8.90; range = 18–61). Most participants were women (80.2%) and from Guatemala (53.7%). The average time participants had been living in Cincinnati was 12.79 years (*SD* = 6.38; range = 1–32). Additional sample demographics are shared in Table 1.

**Table 1.** Survey participant demographics

		*n*	% among respondents to each question
Sex			
	Female	97	81.5
	Male	22	18.5
Age			
	18–25	10	9.0
	26–39	60	54.1
	40–49	31	27.9
	50–59	9	8.1
	60+	1	0.9
Partnership status			
	Married	59	50.0
	Single	30	25.4
	Partnership	22	18.6
	Widowed	4	3.4
	Divorced	3	2.5
Household size			
	1–2	10	8.6
	3–4	40	34.2
	5+	67	57.3
Health insurance status			
	Insured	29	25.2
	Uninsured	86	74.8
Country of birth			
	Guatemala	65	54.6
	Nicaragua	23	19.3
	Mexico	15	12.6
	Ecuador, Venezuela, Colombia, or Peru	9	7.5
	Honduras or Panama	4	3.3
	Dominican Republic or Prefer Not to Say	3	2.5
Time in Cincinnati			
	0–5 years	19	16.8
	6–10 years	26	23.0
	11–20 years	58	51.3
	>20 years	10	8.9
Language(s) spoken at home			
	Spanish only	56	47.9
	Spanish and Mam	32	27.4
	Spanish and English	25	21.4
	Spanish and Mam and English	2	1.7
	Mam only	1	0.9
	English only	1	0.9
Were you employed during the last 6 months?			
	Yes	70	62.0
	No	43	38.1
Do you have children?			
	Yes	105	87.5
	No	15	12.5
How many children are living in your home?			
	0	12	10.6
	1+	101	89.4

*Note. n* = Number of participants. Total unique participants in survey *n* = 121.

### Survey development

In a planning meeting in November 2020, the co-researchers shared that a verbally administered survey would be the best way to achieve our study aim, particularly in the context of public health restrictions. We had four virtual team sessions through the end of December 2020 to develop the survey and review survey administration techniques. The order, flow, wording, and length of Likert scales were co-created with the co-researchers. The instrument was developed in Spanish, and to increase trust, all questions were optional. A co-researcher who spoke Mam would clarify questions in Mam when needed during data collection, but because this is not a written language we did not have a written instrument in Mam. See supplemental materials for English and Spanish versions of the full instrument.

### Survey measures

#### Impact of pandemic on daily life

Experiences during the pandemic were assessed using a modified version of the second and third questions of the Pandemic Stress Index (PSI) tool, a three-question scale that was translated into Spanish using translation, back translation, and comparison/reconciliation (Harkness et al., 2020). This tool was selected from among the few available existing tools during the study design phase by our co-researchers for its relevance and brevity. Our co-researchers modified response options to match local verbiage and experiences (e.g., worry about children’s education). See Table 2 for the questions and response options.

**Table 2.** Survey responses

		*n*	% among respondents for each question
What was the impact of COVID–19 on your daily life?			
	None	3	2.5
	A little	37	31.4
	Moderate	30	25.4
	A lot	25	21.2
	Extreme	23	19.5
Which of the following are you experiencing (or did you experience) during COVID–19 (coronavirus)? (check all that apply)			
	Worrying about friends, family, partners, etc.	111	91.7
	Fear of getting COVID–19	98	81.0
	Fear of giving COVID–19 to someone else	80	66.1
	Frustration or boredom	76	62.8
	Personal financial loss	71	58.7
	More anxiety	61	50.4
	Concern about the virtual education of children (e.g., low grades, poor concentration, class online)	59	48.8
	Getting emotional or social support from family, friends, partners, a counselor, or someone else	59	48.8
	Stigma or discrimination from other people	56	46.3
	Feeling that I was contributing to the greater good by preventing myself or others from getting COVID–19	55	45.5
	More depression	48	39.7
	Confusion about what COVID–19 is, how to prevent it, or why social distancing/isolation/ quarantines are needed	44	36.4
	Other difficulties or challenges	39	32.2
	More sleep, less sleep, or other changes to your normal sleep pattern	35	28.9
	Being diagnosed with COVID–19	25	20.7
	Loneliness	24	19.8
	Problems with paying for virtual education (e.g., computers, internet)	12	9.9
	Not having enough basic supplies (e.g., food, water, medications, a place to stay)	7	5.8
	Change in sexual activity	6	5.0
	Getting financial support from family, friends, partners, an organization, or someone else	5	4.1
	Increased alcohol or other substance use	2	1.7
During the pandemic, have you experienced stress?			
	None	15	12.5
	A little	60	50.0
	Moderate	16	13.3
	A lot	18	15.0
	Extreme	11	9.2
Is this level of stress higher than before the pandemic?			
	Yes	52	61.9
	No	32	38.1

*Note. n* = Number of participants. Total unique participants in survey *n* = 121.

#### Stressors and stress levels

To assess stress, participants were asked to self-describe their stress level using a five-point Likert scale (1 = *Not at all* to 5 = *Very much*). If the participant answered that they had experienced any stress, changes to their stress level were assessed with the follow-up question, “Was your stress level higher than before the pandemic?” (Yes/No).

#### Stress management

The co-researchers were interested in understanding the strategies participants used to manage stress. They developed a list of common strategies based on a previous LU-Salud study and the preferences of their social networks (see Table 3) (Jacquez et al., 2019). In the survey, participants were asked to select each technique they used during the pandemic from this list. They were also asked whether they were interested in learning more about stress and stress management. If they indicated interest, they were asked which options they would be interested in during and after the pandemic from a list generated by the co-researchers.

**Table 3.** Stress management preferences

		*n*	% of total respondents for each question	*X* ^2^
How have you managed stress during this time? (choose all that apply) (*n* = 103)				
	Cleaning or organizing the house	87	84.5	0.69
	Calling close friends	70	68.0	5.22*
	Cooking or following recipes	69	67.0	2.38
	Listening to music	69	67.0	3.66
	Praying	68	66.0	6.00*
	Watching a movie	66	64.1	12.36***
	Playing with your kids	64	62.1	155.94***
	Walking	51	49.5	1.89
	Laughing	46	44.7	24.36***
	Exercising	41	39.8	5.20*
	Looking up information on your phone	39	37.9	19.66***
	Reading	38	36.9	5.17*
	Reading the Bible	33	32.0	0.16
	Dancing	30	29.1	9.81**
	Singing	28	27.2	7.47**
	Spending time with your pets	23	22.3	15.94***
	Deep breathing exercises or meditating	14	13.6	7.05**
	Talking to a psychologist	2	1.9	0.20
Are you interested in learning more about stress management? (*n =* 117)				
	Yes	56	47.9	0.41
	No	61	52.1	
How would you like to learn more about stress management during the pandemic? (choose all that apply) (*n =* 54)				
	Watching YouTube™ videos	31	57.4	4.52*
	Virtual professional–led groups	26	48.1	2.04
	Group text messaging	22	40.7	8.05**
	In–person professional–led groups	10	18.5	8.77**
	In–person peer–led groups	8	14.8	3.04
	Virtual peer–led groups	3	5.6	0.18
How would you like to learn more about stress management after the pandemic? (choose all that apply) (*n =* 51)				
	Watching YouTube™ videos	24	47.1	5.04*
	Virtual professional–led groups	11	21.6	0.00
	Group text messaging	19	37.3	11.08***
	In–person professional–led groups	19	37.3	13.76***
	In–person peer–led groups	7	13.7	4.88*
	Virtual peer–led groups	3	5.9	0.18

*Note. n* = Number of participants who chose at least one response for each survey question. Stress management preference questions were only asked to those who answered “yes” to wanting to learn more about stress management. *X^2^* = Differences between those from countries besides Guatemala and those from Guatemala: *p <. 05 **p < .01, ***p < .001.

### Quantitative analytic approach

Prior to conducting our main analyses, we screened the data for missing variables. Most (74.4%) participants gave complete demographic information. Analysis of missing data across all variables indicated that 1.27% of all items for all participants were missing, and 80% of the items were not missing data for any participant. Approximately 51% of participants had no missing data. According to Little’s MCAR Test, the data appeared to be missing at random, χ2(1,371, *N* = 121) = 1,302.20, *p* = .91. Given the random nature of missing data and minimal levels of missingness, pairwise deletion was applied to all analyses, except for the mediation analysis where the full information maximum likelihood method was used (Kang, 2013).

To complete our main analyses, Pearson chi-square tests were used to assess the differences in COVID-19’s impact on daily life (*none/very mild/moderate* or *a lot/extreme*) by the demographic variables: sex, age, partnership status (*single/widowed/divorced* or *married/partnered*), children at home (*none* or *one or more*), country of birth (*Guatemala* or *All other countries*), household language (*[English and Spanish and/or Mam]* or *[Spanish and/or Mam]*), and employment in the last 6 months (*yes* or *no*). Pearson chi-square tests were also used to analyze differences in stress levels (*none/mild/moderate* or *a lot/extreme*) and stress change (*higher than pre-pandemic* or *not higher than pre-pandemic*) by all demographic variables listed above.

Given the demographics of our sample and impression among our LU-Salud co-researchers that the experiences of Guatemalan-born Latinx immigrants, many of whom have indigenous ancestry, are different, we examined participant differences by country of origin (Guatemala vs. all other countries). Prior LU-Salud work has identified differences in experiences between Cincinnati-based Mexican and Guatemalan immigrants (Zhen-Duan et al., 2017; DiMascio et al., 2020). Our sample size limited our ability to detect significant demographic differences among our Guatemala-born participants compared to other participants, but we nevertheless knew it was important to honor this request and examine differences in pandemic experiences and stress management preferences among Guatemala compared to the rest of the group. We used Pearson chi-square tests to examine significant differences in participant experiences during the pandemic, stress management techniques used during the pandemic, interest in learning stress management techniques, and preferences for techniques during and after the pandemic by country of origin.

A one-way ANOVA with Tukey’s post hoc analysis was used to examine differences in COVID-19’s impact on daily life by time spent in Cincinnati (*0–5 years* vs. *6–10 years* vs. *11–20 years* vs. *20+ years*). We also completed a post hoc mediational analysis to examine the mediating effects of household language on post-pandemic stress change by country of origin. We used JASP Structural Equation Modeling and bootstrapping techniques (1,000 Bias-corrected replications) to estimate the direct and indirect effects of these associations. All statistical analyses were completed in IBM SPSS 28 and JASP. The *p*-value was set to *p* < .05 for all analyses.

### Community conversation procedures and participants

Due to complications from the COVID-19 pandemic, the community conversation (CC) initially needed to be conducted virtually, but most survey participants were uninterested in or unable to do a virtual focus group. Thus, to conduct the CC, we collaborated with a local church whose congregation agreed to host the research team in the spring of 2022 when it was more safe to convene. Twenty-five Latinx adult congregation members participated in the CC and received a $25 gift card. Demographic information of CC participants was not gathered, but the research team observed that, compared to survey participants, the CC had more participants over the age of 50 and more men. No congregation members had participated in the survey. CC participants were served refreshments, shown a presentation of the survey key results and participant demographics, and asked about their impressions and interpretations of the results. The conversation was audio-recorded and transcribed in Spanish using Microsoft Word, with an academic team member checking the transcription for accuracy and correcting it when necessary.

### Qualitative analytic approach

The Spanish transcript of the CC was analyzed using thematic analysis (Braun and Clarke, 2006). Two Spanish-English bilingual academic team members (one of whom is a Latinx second-generation immigrant) familiarized themselves with the data and generated initial codes, which were organized into a codebook. Next, those two team members and one of the LU-Salud co-researchers used the codebook to code the entire transcript. The principal investigator reconciled disparate codes through discussion. Codes were organized into themes, and representative quotes for each theme were translated into English for dissemination.

## Results

### Impact of COVID-19

While 31.4% of participants reported that COVID-19’s impact on their lives was “a little”, 40.7% of participants reported the impact was “a lot” or “extreme.” The majority (91.7%) of participants reported “worrying about friends, family, partners, etc.” “More anxiety” was experienced by more than half of participants (50.4%), and “stigma or discrimination from other people” was also common (46.3%). See Table 2 for additional descriptive statistics on the impact of COVID-19.

Time in Cincinnati was significantly associated with COVID-19 impact (F(3,108) = 3.77, *p* = .013). A Tukey’s post-hoc analysis revealed that those who spent 6–10 years in Cincinnati and those who spent 11–20 years in Cincinnati significantly varied from one another, with those living in Cincinnati for 6–10 years reporting higher impact of COVID-19 on daily life than those living in the city 11–20 years (.29622, 95% CI [.0009,.591], *p* = .049). No other demographic variables were associated with COVID-19 impact.

Those who identified as Guatemalans were more likely to experience stigma during the pandemic (*X* ^2^ [1, *N* = 119] = 8.64, *p* < .01) than those from all other countries. Contrastingly, those who identified as being from other countries were more likely to experience sleep changes (*X* ^2^ [1, *N* = 119] = 16.71, *p* < .001), financial loss (*X* ^2^ [1, *N* = 119] = 17.67, *p* < .001), and financial support (*X* ^2^ [1, *N* = 119] = 6.28, *p* = .01) than Guatemalans.

### Stress levels during the pandemic

As seen in Table 2, many participants reported feeling “a little” (50.0%) stress during the pandemic, and nearly a quarter (24.2%) reported “a lot” or “extreme” stress. Of those who reported stress and compared their stress levels to before the pandemic, more than half (*n* = 52 of 84, 61.9%) reported that their stress level was higher than before the pandemic.

No demographic group (by sex, age, partnership status, parenting status, country of origin, time in Cincinnati, or household language[s]) was more likely to report “a lot” or “extreme” levels of stress than others. However, Guatemalans were less likely to report increases in stress levels during the pandemic than participants from other countries (*X* ^2^ [1, *N* = 83] = 12.64, *p* < .001). Those who did not speak English at home were also less likely to report increases in stress levels during the pandemic than those who did (*X* ^2^ [1, *N* = 81] = 5.53, *p* = .02). No other demographic variables were associated with changes in stress level during the pandemic.

**Figure 2.**
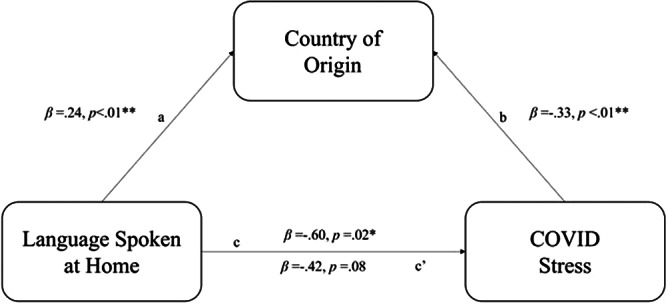
Mediation model of household language on COVID-19 stress through country of origin.*Note: p* < .05*; *p* < .01**. Standardized regression coefficients are displayed. The total effect of language spoken at home on COVID-19 stress is displayed above the arrow; the direct effect of this relationship is displayed below the arrow.

To assess a post hoc hypothesis that country of origin mediates the association between household language and post-pandemic stress change, the direct and indirect effects were estimated with JASP Structural Equation Modeling and bootstrapping techniques (1,000 Bias-corrected replications). As shown in Figure 2, in the mediated model, the indirect effect of language spoken at home on COVID-19 stress through origin country was significant, *β* = −.19 (*SE* = .09; 95% CI = −.42 to −.04; *p* = .04). The direct effect of language spoken at home on COVID-19 stress was not significant, *β* = −.42 (SE = .24; 95% CI = −.83 to.07; *p* = .08), suggesting full mediation.

### Stress management preferences

The most common responses for stress management techniques utilized during the pandemic were “cleaning or organizing the house” (84.5%), “calling close friends” (68.0%), “cooking or following recipes” (67.0%), and “listening to music” (67.0%). The least common responses were “talking to a psychologist” (1.9%) and “deep breathing exercises or meditating” (13.6%).

A series of chi-square analyses (*X* ^2^ [1, *N* = 119]) showed that individuals from countries besides Guatemala were more likely than Guatemalans to use the following stress management strategies: deep breathing, praying, talking to a friend, singing, dancing, exercising, watching a movie, laughing, reading, spending time with family, spending time with pets and looking for information on their phones related to fitness and/or entertainment. See Table 3 for chi-square and *p*-values.

Chi-square analyses (*X* ^2^ [1, *N* = 119]) further revealed no difference in interest in learning more about stress/stress management by country of origin. Among participants who were interested in learning more, Guatemalans were less likely to prefer learning through in-person groups led by professionals and more likely to prefer learning through text messaging groups and YouTube videos *during* the pandemic. *After* the pandemic, Guatemalans were less likely to prefer learning through in-person groups led by peers and in-person groups led by professionals, while they were more likely to prefer learning through text messaging groups and YouTube videos. See Table 3 for chi-square and p-values.

### Community conversation interpretation of survey results

Qualitative data from the CC revealed that the experiences and stress levels of Latinx immigrants in Cincinnati were different based on demographics (see Table 4). Overall, many CC participants were surprised that stress levels reported by survey participants were not higher, given their personal experiences. They noted that some groups within the community were disproportionately impacted by the pandemic (e.g., older individuals with chronic medical conditions, school-age children and their parents). They also described grief over social distancing restrictions due to the cultural importance of community, family, and worshiping together. Despite the importance of community, CC participants were not entirely surprised that private ways of learning about stress management were preferred but also acknowledged that the resumption of peer-led women’s support groups was helping reduce stress.

**Table 4.** Community conversation themes and representative quotes

Community		
The importance of community and family	#2: …as a congregation, as Christians, it affected us “quite a bit”. Because we could not come together how we are right now and for us as people that come together constantly worshipping and praising God, serving God in the community on top of evangelizing in talking with people #22: For me, it’s surprising that the majority replied, “a little”. I would think quite a bit especially in our Hispanic culture as we are used to being around people, visiting family and friends.	#2: … como feligreses como cristianos que nos afectó “bastante”. Porque no pudimos reunirnos como estamos ahorita y eso para nosotros las personas que nos reunimos constantemente alabando y adorando a Dios y sirviendo a Dios en la comunidad a través de evangelizar de hablar con las persona #22: A mí me sorprende que la mayoría fue “un poco”. Yo pensaría que bastante especialmente en–en nuestra cultura hispana nosotros estamos acostumbrados a estar rodeados de personas a visitar a familiares a nuestros amigos amistades
The importance of privacy about struggles	#18: if I’m stressed maybe I’m not going to want other people to know that I’m feeling stressed, even though I’m struggling with it. So, maybe I’d prefer to watch a virtual group #18: Yes, maybe for the same reason of privacy because it’s not looked well upon to share and–and as we said it’s not about just dealing with stress but someone who is suffering with stress and generally they do not want those around them to know. And when they go ask for help from a counselor in confidence, they do not share this with friends, for example.	#18: si yo tengo estrés tal vez no voy a querer que la gente sepa que tengo estrés, aunque sufro del estrés. Entonces tal vez prefiero ir a un vídeo que a un grupo virtual #18: Sí tal vez por la misma privacidad porque no–no es algo agradable para compartir y–y digamos o sea lo que decía ahorita no es el que está estresado hoy está sufriendo por estrés no quiere que su entorno lo sepa generalmente. Y cuando van a pedir ayuda van a un consejero de confianza no comparten eso con sus amigos, por ejemplo.
Experiences during the pandemic		
Difficulties and challenges during the pandemic	#20: [For those who answered] “quite a bit” could have been that maybe they lost their jobs and had no ways to provide for their family. #12: … It was scary to go to the store without even putting on a mask, it was scary to visit someone or that someone would visit us. As soon as we’d meet someone at the door, we’d throw on a mask because of this fear. So, this fear produces stress and I believe that 24 h a day, we were battling this fear. #20: Something I noticed was the academic level for the kids, uh, I believe they had quite a bit of stress as they had to go from in–person to virtual and this transition was horrible. Many parents could not connect their children with computers. Many aren’t familiar with technology and the teachers had a lot of trouble as well…I think the repercussions will last for years… #2: … And we can hear the level of stress and frustration because some of their benefits have stopped, or they did not receive services	#20: “bastante” pudo haber sido que tal vez perdieron sus trabajos y no tenían formas como proveer para su familia. #12: Era un temor ir al mercado, era un temor ir a visitar a alguien o que alguien nos visitara. Inmediatamente cuando recibíamos alguien en la puerta, pero tenemos que estar con la boca tapada porque qué tal si verdad es un temor. Y entonces ese temor produce el estrés yo creo que las 24 horas del día por un tiempo estuvimos batallando con el tema #20: Algo que noté fue de que en el–el nivel del estudio de los niños, ah, creo bastante estrés porque se tuvieron que ir de presencial a virtual y fue horrible esa transición. Muchos padres no podían conectar a sus niños a las computadoras. Muchos no están familiarizados con la tecnología y los maestros tuvieron muchos problemas también…Y lo pienso que esto va a repercutir varios años… #2: … Y sí es podemos escuchar el estrés que ellos tienen y la y la cómo se dice la frustración porque eso les causa porque algunos de ellos suceden sus beneficios fueron de los pararon no recibieron o no han recibido servicios
Support and survival during the pandemic	#19: I think that some of the reasons were because there was a lot of help [and services] in the community and also some were able to continue working. #16: … I do not know who I’m going to ask a favor from, we’ll have to congratulate you because when that information came that hurt us, which part of the government were the entities that began to calm us down, gave us good information, very good recommendations until we could sneeze –all of that was worth it…I even remember a cartoon – it is better to be locked up than not in a hospital, it is better to be in the house than in a grave and all that calmed us down a lot.	#19: Yo pienso que alguna de las razones fue porque hubo ayuda mucha ayuda en la comunidad y también este algunos pudieron seguir trabajando. #16: No sé a quién le voy a pedir favor a ustedes tendremos que felicitar porque cuando vino esa de esa información que nos hacía daño de que parte del gobierno quienes fueron las entidades que empezaron a calmarnos a darnos buena información muy buenas recomendaciones hasta poder estornudar –todo eso valió la pena… Recuerdo hasta una caricatura – es mejor estar encerrado que no en un hospital es mejor estar en la casa que no en una tumba y todo eso nos calmó bastante.
Adaptation was necessary; comparing experiences during the pandemic to before the pandemic	#22: I think [they said] “a lot” because many of our parents had to adapt to a new system…of technology during the pandemic and we are used to being in person. But to be in person, we had to adapt ourselves to a new way of interacting with each other.	#22: yo creo que fue “bastante” porque también muchos de nuestros padres a él el adaptarse a un nuevo sistema…de tecnología y durante la pandemia y estamos nosotros impuestos estar en persona, pero hacer las cosas en persona y ya adaptarse a un sistema donde ya no podíamos interactuar.
Differences in pandemic experiences		
There were differences in experiences between Latinx immigrants and others	#18: I think that a large part of the priority of foreigners is work as such and for Latinos, many of the jobs, in other words, what is done here in the United States has to do with essential jobs that were never good.. They were affected very little so maybe it did not affect them.	#18: Creo que gran parte de la prioridad de los extranjeros es el trabajo como tal y por los Latinos muchos de los trabajos eh en cuanto ósea lo que lo que se hace aquí en Estados Unidos tiene que ver con los trabajos de esenciales que nunca se dieron bueno. Se vieron afectados muy poco entonces tal vez no les afectó
There were differences in experiences for subgroups within the Latinx immigrant community	#19: I think that one of the reasons was because there was a lot of help in the community and also some were able to continue working. That would be a bit going a long way because some of the aid was only for those who were documented, those who were not undocumented did not receive that aid. #8: I see that the majority were Guatemalans, right? So, it’s because as Guatemalans the country is developing and there exists lots and lots of suffering and many things happen. So, I think they are prepared to handle this more than another person that’s not at this level. #16: I go with “quite a bit” because of us from the older days…We had a problem with medical appointments and we were not attended to and it was incredible when we ran out of medicine for high blood pressure for diabetes and there was no appointment	#19: Yo pienso que alguna de las razones fue porque hubo ayuda mucha ayuda en la comunidad y también este algunos pudieron seguir trabajando. Eso sería un poco yendo bastante porque algunas de las ayudas eran nada más para los que estaban documentados los que no estaban indocumentados no recibieron esa ayuda. #8: Veo que la mayoría que fueron Guatemaltecos ¿no? Entonces eso de ver a Guatemaltecos como es un país que está desarrollando existe mucho sufrimiento y pasan muchas cosas. Entonces pienso yo que están preparados en eso que tiene que lo enfrentan mejor que otra persona que no está a ese nivel #16: Yo voy por “bastante” porque especialmente los días viejitos como nosotros…Tuvimos problema con las citas médicas ya no fuimos atendidos y era increíble se nos acababa la medicina para la presión alta para la diabetes y no había cita por ahí

CC participants suggested that survey participants did not describe overall higher stress levels because they had been reflecting on services and resources available during the pandemic, and because many Latinx immigrants having jobs considered essential was protective against job loss. They also noted that some Central Americans, particularly Guatemalans, had experienced high levels of suffering and stress prior to coming to the U.S. and within the U.S. prior to the pandemic, and therefore may not have noticed a dramatic increase from an already-high stress level. One of our co-researchers emphasized that the local Guatemalan immigrant community was deeply affected by the pandemic and cautioned that survey results showing no increase in stress levels do not suggest that service providers do not need to serve or worry about the impact of stress on Guatemalans.

## Discussion

The COVID-19 pandemic has disproportionately affected the Latinx community in the U.S., and our results show that in an NTID, the pandemic impacted subgroups of the Latinx immigrant community differently. In our sample, those who did not speak English at home were less likely to report increases in their stress levels during the pandemic. Across nationalities of our sample, Guatemalans were more likely to report experiencing stigma yet less likely to report increases in their stress level since pre-pandemic. They were also less likely to report using many coping strategies commonly used by other local Latinx immigrants. Guatemalans in our sample may have had higher baseline levels of stress prior to the pandemic and experienced or described stress differently than other Latinx groups, particularly because 28.9% of survey participants reported speaking Mam (an indigenous language in Guatemala) at home. Our CC participants referred to the “suffering” of Guatemalans before migration, and while much of Central America experienced conflict, civil war, and human rights abuses in the 1960s–1990s, the war in Guatemala was the longest and had the most casualties, with indigenous Guatemalans being disproportionately affected. Today, about 42% of Guatemalans are indigenous (the highest in Latin America), are 1.7 times more likely to be living in poverty than non-indigenous Guatemalans, and also more likely to be living in poverty than other indigenous groups in Latin America (World Bank Group, 2016).

A telephone study completed during the pandemic in Guatemala showed similar levels of stress as our results (greater than 60% of their sample denied stress, while 62.5% of our participants reported no or “a little” stress. Our CC participants expressed surprise that levels of stress were not higher but acknowledged that Guatemalans may have different historical experiences and baseline stress levels compared to other Latinx immigrants. The finding that a sense of privacy around struggling was valued could additionally contribute to the lower-than-expected levels of reported stress by survey participants. Available research shows that Guatemalans exposed to war have increased rates of mental disorders, particularly when social networks are disrupted (Herrera et al., 2005; Puac-Polanco et al., 2015; Kowal et al., 2020).

More survey participants reported increased anxiety (50.4%) than moderate or higher levels of stress (37.2%). Similarly, in the Guatemalan phone survey, more people reported anxiety during the pandemic (46%) than stress (36%). Therefore, it is possible that culturally specific perceptions of “anxiety” and “stress” may have affected self-reported levels of stress in our survey. Prior research by our study team illustrates that local Guatemalan immigrants were less likely than local Mexican immigrants to report stress as a priority health concern, which may suggest different perceptions (DiMascio et al., 2020).

Across demographic characteristics, we found that no group was more likely to report “a lot” or “extreme” levels of stress than others. These findings contrasted with prior literature, which highlights the unique susceptibility of women, those who are unmarried, and those who have children to stress and mental health outcomes during the pandemic (Kowal et al., 2020; Gomez-Aguinaga et al., 2021). However, our findings may reflect limitations in our sample size and diversity, which could compromise statistical power to detect within-group differences. We are not able to compare stress levels in this group to non-Latinx Cincinnatians with our available data, but recognize that structural inequities and racism contribute to the socioeconomic disparities faced by Latinx Cincinnatians. With a larger sample size, we might be able to better explore ethnic and sociodemographic factors within the Cincinnati Latinx community that contribute to stressors and stress.

### Impact of the pandemic

Most (56.8%) participants reported that the impact of the pandemic on their daily lives was “a little” or “moderate.” This is in contrast with data showing that in other NTID states with less well-established Latinx communities such as Oregon, Washington and Utah, there were higher rates of cases and less support systems in place than in states with more well-established Latinx communities such as California, Arizona or New Mexico (Jordan and Oppel, 2020). The most common pandemic-related experiences reported by our participants were worrying about others, which has also been a theme in qualitative studies and may be higher than self-concern in Latinx immigrant groups (Moyce et al., 2021; Quandt et al., 2021). Reports of “stigma or discrimination from other people” were also common in our sample (46.3%) as has been described in other studies during the pandemic, and may reflect racism-related stress in this NTID (Hearst et al., 2021). Substance use was least likely to be described, in contrast with other reports that show that the pandemic may have contributed to immigrants’ stress and substance use (Romano and Sánchez, 2023). As pointed out by a CC participant, it is possible that the reported level of impact on the lives of Latinx immigrants in Cincinnati is blunted because many immigrants in our NTID have low-income and essential jobs; this is supported by local labor force data showing that the industries in which most Latinx Cincinnatians work are manufacturing, administrative, support, waste management, accommodation and food services (Economics Center Research and Consulting, 2022). Lastly, given that a central theme was keeping struggles private and the stigma reported by our participants, it is possible that survey participants were endorsing the impact of the pandemic on their experience, particularly given the surprise expressed by CC participants at survey results. The result that non-Guatemalans were more likely to experience both financial loss and support may suggest the presence of community support, which was also a key qualitative theme but should be explored more to understand the experiences of those from countries other than Guatemala that confer socioeconomic disadvantage.

### Stress management preferences

The current study showed that asynchronous, flexible methods of learning about stress management (watching YouTube videos or being a part of group texts) were preferred and that once public health restrictions were lifted, professional-led groups were preferred over peer-led groups. Contrary to this, one of our CC participants expressed surprise at these results given the importance of a peer-based women’s group that her church organized. Other research has shown that faith-based health promotion programs are a promising stress management approach for the Latinx community, and pre-pandemic research shows that peers or “promotoras”/“compañeras” are a preferred source of information (Schwingel and Gálvez, 2016; Nápoles et al., 2018; Jacquez et al., 2019; Moyce et al., 2021). Compared to previous studies in which physical activity was a preferred method of stress reduction, cleaning the house was a preferred stress management strategy in this study, likely in part because people were spending more time in their homes (Jacquez et al., 2019). Connecting with others was the second-most common currently used method of stress management, which aligns with CC qualitative themes showing the importance of community and grief associated with restrictions on gathering and with other qualitative work during the pandemic (Moyce et al., 2021). Given that survey participants demonstrated a preference for professional-led groups, but “talking with a psychologist” was the lowest-rated stress management tool being used, our results show that there is a critical need for psychotherapists who serve the Latinx community in Cincinnati. Given the lack of Latinx and Spanish-speaking psychotherapists in Cincinnati, the need may be better met if those professionals were supported to integrate into existing community-based initiatives such as groups hosted by churches or social service organizations, or focusing efforts on text-based support or video-based education.

### Limitations and future directions

There are a few limitations of this work to note when reviewing these findings. Many survey items were generated by our research team to preserve local relevance, which resulted in a survey with limited available psychometric properties. A few questions used a “select all that apply” response system without an option for participants to indicate “none”; in these cases, missing responses were coded as denial rather than true missingness. Thus, despite the participant response rate being relatively high across survey items, results may be biased towards negative responses due to conflation of negative and missing responses. Ideally, our CC would have been hosted with survey participants and hosted close to the time of survey data collection, but despite efforts to do so there were no survey participants who expressed interest in virtual or in-person group follow-up, and so the reflections of the CC were in a different phase of the pandemic. We are grateful to the local church which hosted our CC, but recognize that their interpretation of our results is more useful than only our own, but may be particular to the religious and geographic community served by the partner church. Specifically, we are eager to offer more insight into the differential experiences of the Guatemalan survey participants compared to those born elsewhere, but we are not able to do so given our inability to re-engage survey respondents. Also, given the Guatemalan and Nicaraguan backgrounds of our community co-researchers, data collection occurred predominantly in ethnic enclaves of these subgroups, which may have limited generalizability. Finally, our predominantly female, married, and with children participant pool left us underpowered to complete extensive within-group comparisons.

Despite these limitations, this work provides important reminders about the heterogeneity of the Latinx immigrant population in the U.S. and about the vulnerability and resilience of the community. Future research should consider the use of cultural consensus modeling to explore the concepts of anxiety and stress among Guatemalans. In addition, further exploration of the differences in experiences within Latinx immigrant groups, particularly in NTIDs, would help to clarify psychosocial needs in subgroups. Specifically, future research by our group would prefer to be able to distinguish the experiences of Latinx community members based on all countries of origin and/or based on the timing of immigration given that many community members beyond Guatemalans have experienced significant and distinct hardship before, during, and after immigration.

## Supporting information

Stryker et al. supplementary material Stryker et al. supplementary material

## Data Availability

The dataset supporting the conclusions is available from the corresponding author on reasonable request.

## Supplementary material

The supplementary material for this article can be found at http://doi.org/10.1017/gmh.2024.101.
