# Supplementary material for: Stress management preferences and stress experiences among Latinx immigrants in the United States during the COVID-19 pandemic: Mixed-methods results from a community-academic research team
Source: Glob Ment Health (Camb). 2024 Oct 23;11:e94. doi: 10.1017/gmh.2024.101 (PMC11504930; doi:10.1017/gmh.2024.101)
Supplement: Stryker et al. supplementary material [file S2054425124001018sup001.pdf]

LU-SALUD CUESTIONARIO

Pregunta de investigación: ¿Para los Latinos viviendo en Cincinnati, cómo se debería proporcionar los servicios comunitarios para el manejo del estrés durante y después de la pandemia?

Preguntas Demográficas

1. Sexo ....Masculino / Femenino
2. Edad ...._______
3. Estado civil ....Casado / Soltero / Unión Libre / Divorciado / Viudo
4. ¿Cuantas personas viven en su casa?.._______
5. ¿Tiene hijos?.. Sí / No
6. ¿Numero de hijos en su casa? ...._______
7. ¿País de origen? ....__________________
8. ¿Tiempo viviendo en Cincinnati? ....__________________
9. ¿Lenguaje hablado en casa? ....Inglés / Español / Mam / Otro idioma:_________
10. ¿Durante los últimos seis meses, trabajaba? ....Sí / No
11. ¿Vive en casa o apartamento? ....Casa / Apartamento
12. ¿En que vecindario vive?.._________________________
13. ¿Tiene seguro? ....Sí / No
14. ¿Sus hijos tienen seguro? ....Sí / No
15. Sús ingreso anuales son.........menor a $30,000 / mayor a $30,000 / prefiero no responder

16. ¿Cual es/ fue el impacto de COVID-19 (coronavirus) en su vida cotidiana? *(marque una "x")*

    ___ Nada
    ___ Un poco
    ___ Bastante
    ___ Mucho
    ___ Muchísimo

Preguntas acerca del estrés

17. **¿Cual de las siguientes experiencias estas pasando ahora (o has pasado) durante COVID-19 (coronavirus)?** *(Marque todas las que apliquen con una "x")*

___ Fui diagnosticado con COVID-19
___ Miedo de contagiarme con COVID-19
___ Miedo de contagiar a otros con COVID-19
___ Preocupación por amigos, familia, compañeros/as, etc.
Si su respuesta es Sí:
____ En su zona local
____ en otras partes de los Estados Unidos
____ Afuera de los Estados Unidos
___ Estigma o discriminación de otras personas (es decir, que la gente lo trate diferente por su identidad, por tener síntomas, u otros factores relacionados con COVID-19)
___ Pérdida financiera personal (por ejemplo, pérdida de su sueldo, pérdida de ingreso, perdida de ahorros, menos envió de remesa familiar, cancelaciones relacionadas a viajes, inhabilidad de pagar la renta/el alquiler)
___ frustración o aburrimiento
___ No tener provisiones básicas (es decir, comida, agua, medicamentos, un lugar donde quedarse)
___ más ansiedad
___ más depresión /deprimido
___ dormir más, dormir menos, u otros cambios en su rutina normal de dormir
___ más uso de bebidas alcohólicas o substancias ilícitas
___ un cambio en actividad sexual (si su respuesta es Sí—¿incrementó o se redujo?)
___ soledad
___ confusión sobre COVID-19, como prevenirlo, o porque la distancia social, el aislamiento y la cuarentena son necesarios
___ la sensación que estaba contribuyendo al bien común previniendo que yo u otros nos enfermásemos con COVID-19
___ recibiendo apoyo <u>emocional o social</u> de la familia, amigos, compañeros, consejero/a, u otra persona
___ recibiendo apoyo <u>financiero</u> de la familia, amigos, compañeros, una organización u otra persona
__ problemas con el pago de la educación virtual (internet, computadoras)
__preocupaciones con el aprendizaje de los hijos (clases en línea, notas bajas, falta de asistencia, falta de concentración)
___ otras dificultades o retos
¡Queremos escuchar de usted! Por favor comparta con nosotros:

____

18. ¿Cuáles son los tres desafíos mas importantes que han impacto a su vida (de la lista anterior)? *Marque el uno, dos, y tres especificado abajo (orden cronológico de los tres desafíos mas importantes)*

1.

2.

3.

Preguntas acerca del acceso al internet o el teléfono

19. ¿Tiene internet en casa? ....Sí / No

20. ¿ ¿Qué usa para acceder a Internet en casa? *(marque todas las que apliquen con una "x")*

___ Teléfono
___ Computadora / portátil
___ Tableta

21. ¿Con qué frecuencia tiene problemas con la conexión para ver o usar los videos? *(marque una "x")*

___ Nunca
___ Raramente
___ A veces
___ A menudo
___ Siempre

22. ¿A cuáles de estos servicios tiene acceso regular? *(marque todas las que apliquen con una "x")*

___ Zoom™
___ Microsoft Teams™
___ Google Meet™
___ Skype™
___ WhatsApp™
___ Facebook™
___ Facebook Messenger™
___ YouTube™
___ Twitter™

Otros: ____

23. ¿En los últimos 6 meses, cuáles de estos servicios utilizaste <u>al menos una vez a la semana</u>?

___ Zoom™
___ Microsoft Teams™
___ Google Meet™
___ Skype™
___ WhatsApp™
___ Facebook™
___ Facebook Messenger™
___ YouTube™
___ Twitter™

Otros: ____

Preguntas acerca del manejo del estrés y las preferencias

24. ¿Durante la pandemia, ha sufrido por el estrés?

___ Nada *(Si la persona marque nada para 24, no hay preguntar el 25-6 y continúe en el 27)*
___ Un poco
___ Bastante
___ Mucho
___ Muchísimo

25. ¿Diría que este nivel de estrés es mas alto que antes de la pandemia?...............Sí / No

26. ¿Como has manejado el estrés durante la pandemia?

___ hacer ejercicio
___ técnicas de respiración o meditación
___ bailar
___ cantar
___ ver una película
___ escuchar música
___ reírse
___ caminar
___ leer
___ rezar
___ leer la biblia
___ hacer llamadas con amistades.familiares
___ cocinar o seguir recetas
___ jugar con los niños
___ pasar tiempo con sus mascotas
___ buscar información acerca de salud o el entretenimiento en teléfonos
___ limpiar o ordenar la casa
___ hablar con una psicóloga

otro (por favor especifique): ___________________________

27. ¿Esta interesado en aprender mas acerca del estrés y el manejo del estrés?.......Sí / No

....*(Si dicen no, aquí termina la encuesta)*

28. ¿Tiene suficiente privacidad en casa para participar en un grupo virtual?...............Sí / No

29. ¿Como le gustaría aprender mas acerca del manejo del estrés durante la pandemia?

___ grupos en persona guiados por sus compañeros
___ grupos en persona guiados por unos Profesionales
___ mirando videos en YouTube™ para aprender más
___ grupos virtuales guiados por sus compañeros
___ grupos virtuales guiados por unos profesionales
___ grupos de mensaje de texto

30. ¿Como le gustaría aprender mas acerca del manejo del estrés después de la pandemia?

___ grupos en persona guiados por sus compañeros
___ grupos en persona guiados por unos Profesionales
___ mirando videos en YouTube™ para aprender más
___ grupos virtuales guiados por sus compañeros
___ grupos virtuales guiados por unos profesionales
___ grupos de mensaje de texto
