# Supplementary material for: Stress management preferences and stress experiences among Latinx immigrants in the United States during the COVID-19 pandemic: Mixed-methods results from a community-academic research team
Source: Glob Ment Health (Camb). 2024 Oct 23;11:e94. doi: 10.1017/gmh.2024.101 (PMC11504930; doi:10.1017/gmh.2024.101)
Supplement: Peer review history

# Editor report: Recommendation: Stress management preferences and stress experiences among Latinx immigrants in the United States during the COVID-19 pandemic: Mixed-methods results from a community-academic research team — R0/PR2

**DOI:** 10.1017/gmh.2024.101.pr2

**Affiliations:** Department of Global Health and Social Medicine, Harvard Medical School, United States

No accompanying comment.

# Editor report: Decision: Stress management preferences and stress experiences among Latinx immigrants in the United States during the COVID-19 pandemic: Mixed-methods results from a community-academic research team — R0/PR3

**DOI:** 10.1017/gmh.2024.101.pr3

**Affiliations:** Johns Hopkins University Bloomberg School of Public Health, United States

No accompanying comment.

# Editor report: Recommendation: Stress management preferences and stress experiences among Latinx immigrants in the United States during the COVID-19 pandemic: Mixed-methods results from a community-academic research team — R1/PR5

**DOI:** 10.1017/gmh.2024.101.pr5

**Affiliations:** Department of Global Health and Social Medicine, Harvard Medical School, United States

No accompanying comment.

# Editor report: Decision: Stress management preferences and stress experiences among Latinx immigrants in the United States during the COVID-19 pandemic: Mixed-methods results from a community-academic research team — R1/PR6

**DOI:** 10.1017/gmh.2024.101.pr6

**Affiliations:** Johns Hopkins University Bloomberg School of Public Health, United States

No accompanying comment.
